# Employing Siamese Networks as Quantitative Biomarker for Assessing the Effect of Dolphin-Assisted Therapy on Pediatric Cerebral Palsy

**DOI:** 10.3390/brainsci14080778

**Published:** 2024-07-31

**Authors:** Jesús Jaime Moreno Escobar, Oswaldo Morales Matamoros, Erika Yolanda Aguilar del Villar, Hugo Quintana Espinosa, Liliana Chanona Hernández

**Affiliations:** 1 Centro de Investigación en Computación, Instituto Politécnico Nacional, Ciudad de México 07700, Mexico; omoralesm@ipn.mx; 2 Escuela Superior de Ingeniería Mecánica y Eléctrica, Unidad Zacatenco, Instituto Politécnico Nacional, Ciudad de México 07738, Mexico; eaguilard2000@alumno.ipn.mx (E.Y.A.d.V.); hquintana@ipn.mx (H.Q.E.); lchanona@gmail.com (L.C.H.)

**Keywords:** Siamese network, biomarker, cerebral palsy, Dolphin-Assisted Therapy, EEG

## Abstract

This study explores the potential of using a Siamese Network as a biomarker for assessing the effectiveness of Dolphin-Assisted Therapy (DAT) in children with Spastic Cerebral Palsy (SCP). The problem statement revolves around the need for objective measures to evaluate the impact of DAT on patients with SCP, considering the subjective nature of traditional assessment methods. The methodology involves training a Siamese network, a type of neural network designed to compare similarities between inputs, using data collected from SCP patients undergoing DAT sessions. The study employed Event-Related Potential (ERP) and Fast Fourier Transform (FFT) analyses to examine cerebral activity and brain rhythms, proposing the use of SNN to compare electroencephalographic (EEG) signals of children with cerebral palsy before and after Dolphin-Assisted Therapy. Testing on samples from four children yielded a high average similarity index of 0.9150, indicating consistent similarity metrics before and after therapy. The network is trained to learn patterns and similarities between pre- and post-therapy evaluations, in order to identify biomarkers indicative of therapy effectiveness. Notably, the Siamese Network’s architecture ensures that comparisons are made within the same feature space, allowing for more accurate assessments. The results of the study demonstrate promising findings, indicating different patterns in the output of the Siamese Network that correlate with improvements in symptoms of SCP post-DAT. Confirming these observations will require large, longitudinal studies but such findings would suggest that the Siamese Network could have utility as a biomarker in monitoring treatment responses for children with SCP who undergo DAT and offer them more objective as well as quantifiable manners of assessing therapeutic interventions. Great discrepancies in neuronal voltage perturbations, 7.9825 dB on average at the specific samples compared to the whole dataset (6.2838 dB), imply a noted deviation from resting activity. These findings indicate that Dolphin-Assisted Therapy activates particular brain regions specifically during the intervention.

## 1. Introduction

Spastic cerebral palsy is the most common form of cerebral palsy, characterized by motor dysfunction due to early brain damage, and manifests itself in various motor impairments, including hypertonia, spasticity, impaired motor coordination, and delayed developmental milestones. The diagnosis involves clinical evaluations, neuroimaging studies, and standardized neurological evaluations. Advances in neuroimaging, such as Magnetic Resonance Imaging (MRI) and Diffusion Tensor Imaging (DTI), have improved early diagnosis and personalized interventions. Furthermore, SCP is the leading cause of childhood disabilities, with a frequency of 2–2.5 per 1000 births in Western countries. For example, France has approximately 130,000 affected individuals. It is estimated that two out of every 1000 newborns will develop cerebral palsy, with about 40% of the cases being severe. Around 10,000 babies and children are diagnosed with the disease each year. Risk factors such as prematurity, low birth weight, maternal-child malnutrition, and inadequate prenatal care contribute to its prevalence.

Given these aetiological risk factors, it is not surprising that some studies suggest that the prevalence of SCP has increased over the last 30 years [1,2]. It has a great influence on the quality of life that burdens healthcare systems worldwide. Knowledge of SCP from the point of view of epidemiology, causes, and treatment is crucial for the purposeful management of results and guiding healthcare policy [3,4].

Himmelmann and Uvebrant in [1] explore the epidemiology of cerebral palsy throughout Europe, focusing on collaborative efforts to understand prevalence rates and demographic factors that influence its occurrence. On the other hand, Chen et al. in [2] study the term and near-term new borns encompassing maternal and perinatal factors that probably increase risk of cerebral palsy. The existing evidence of intervention for improving gross motor performance in children with cerebral palsy and the necessity to individualize therapy are well summarized by Lucas et al. in [3]. Finally, Ahmed et al. in [4] combine the analysis times and partners of interest in Canada and offer a comparative analysis of the global data trends. These studies illuminate the multifaceted nature of cerebral palsy, in particular disparities across populations and lifelong consequences including current practices that guide prevention strategies. Universal, quality healthcare is important to advance individual and global well-being.

On the other hand, SCP is a prevalent condition in the Mexican population, presenting significant challenges in healthcare and quality of life [5,6]. In addition, SCP is a public health problem with limited epidemiological data, but recent research shows an increasing prevalence similar to global trends [7]. Tohen-Zamudio et al. in [7] present prevalence data specific to Mexico, revealing regional epidemiological trends and demographic factors.

Currently available treatment options for affected individuals have been examined in recent studies, which focus on these interventions to improve motor function and quality of life. Concerning research on rehabilitation strategies, one of the studies was carried out by Lerma-Castaño et al. in [8], focusing on the application of physical therapy that is very effective in improving motor quality and daily activity. Similarly, Blumetti et al. in [9] explored the botulinum toxin injected as a treatment for muscle spasticity in patients with SCP, explaining how it had an impact on functional improvement and mobility.

Therefore, Delgado in [10] examined the effects of parental education and support programs on general health of children in Mexico with SCP; it is a kind of nature-oriented intervention and family-based care modality. The findings highlight that informed care provisions could help increase treatment adherence not only in this population but also can further promote a holistic approach for those affected. Furthermore, Ibarra-Rodríguez in [11] examined the possibility of providing rehabilitation services to remote rural communities in Mexico using telemedicine as a method to allow access to specialized care and possible treatments outside traditional clinic settings. Together, these studies highlight a variety of strategies that have been used to address SCP management in Mexico and span traditional therapies and more innovative uses, for example, technology or community-based support.

**Figure 1:**
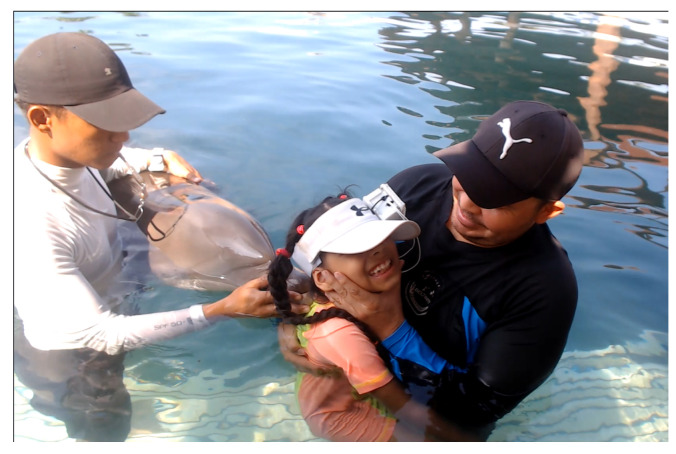
Child diagnosed with spastic cerebral palsy engaging in a Dolphin-Assisted Therapeutic intervention, wherein the tranquil presence of these highly intelligent marine mammals facilitates and enhances their therapeutic process.

In general, parents strive to improve the quality of life of their children. Consequently, optimal treatment of these patients requires a multidisciplinary approach that includes early intervention, rehabilitation therapies, pharmacotherapy, orthopedic interventions, and assistive devices. Early intervention programs, such as physical, occupational, and speech therapy, have shown efficacy in improving motor impairments and functional outcomes, while emerging interventions such as Dolphin-Assisted Therapy show promise, but require further research to confirm their long-term efficacy and mechanisms. Figure 1 shows a child diagnosed with spastic cerebral palsy engaging in a Dolphin-Assisted Therapeutic intervention, wherein the tranquil presence of these highly intelligent marine mammals facilitates and enhances their therapeutic process.

The paper is organized as a whole into five main sections. Section 2 discusses the related work, which provides a holistic understanding of existing methodologies and emphasizes where our approach fits in. In Section 3, we describe our methodology in which data from SCP patients who participated in DAT sessions are used to train a Siamese network to learn commonalities and patterns between pre-therapy and post-therapy evaluations. The architecture of this network enables accurate comparisons in a common feature space and recognition of biomarkers that correspond to the effectiveness of therapy. Section 4 reports the results, proposing encouraging results consisting of unique patterns in the output of the network associated with an improved SCP symptom after DAT. Section 5 summarizes and concludes the potential utility of the Siamese network as a quantitative imaging biomarker to monitor treatment outcomes in children with SCP, providing an objective quantifiable tool to evaluate therapeutic efficacy.

## 2. Related Work

Siamese Neural Networks (SNNs) are a class of neural networks designed to identify similarities between two input items. Unlike traditional neural networks that focus on classification tasks, SNNs are tailored for comparison tasks. They are in fact two copies of the same subnet conducting separate processing on different inputs with their outputs being compared via a similarity metric’s output. They are also very good for problems where the solution needs to discover data similarity or dissidence, i.e., patient records match, diagnosis of (non-)sickness, etc. Below are some of the main applications that benefit from SNN in healthcare, highlighting their versatility and performance (i.e., patient similarity can be learned by using these links): matching records across patients, thereby supporting diagnosis and treatment planning. Using existing patient data records, SNN identifies a new case and suggests the different treatment option that can be used for a similar disease.

The potential benefits of using SNN in healthcare are increased diagnostic accuracy, because a neural network like this learns better by leveraging historical data, providing a data-driven basis for medical decisions. So, the SNN is capable of diagnosing diseases by comparing medical images, e.g., radiographs or magnetic resonances, for detection. From this, SNN can identify similar cases in new patient images by learning patterns and features related to specific conditions. As a result, diagnosis times are faster and more accurate, with less reliance on subjective interpretation by medical professionals.

SNNs have thus far demonstrated immense potential within various healthcare-based use cases and by showing that they can support several effective methods of improving diagnostic accuracy; in that respect, Feng and Chaspari in [12] also explain an application for the deployment of Siamese Neural Networks in healthcare, particularly with patient similarity matching helping to improve clinical outcome by learning from larger datasets.

The result of our study shows that although SNN has high scalability and accuracy, a large amount of training data is necessary to work exactly as it was designed due to its application complexity. In addition, the work by Feng and Chaspari in [12] explores how SNN can be put into practice and used to overcome health service challenges. The authors demonstrate this by showing how SNN can lead to strong and accurate models to compare complex medical data that, in turn, will provide substantial improvements across different healthcare pathways.

In medical areas, SNNs can be used to compare DNA sequences to predict a common gene or its mutation. In this case study, the focus was on a more effective application of genetic disorders, because personalized medicine can be provided. SNN offers a scalable platform that can handle large genomic datasets to identify diagnostic characteristics of rare genetic diseases. Furthermore, SNNs can be used to detail the explanation of the technical implementation of SNNs, including network architecture, training methodologies, and performance metrics. The twin subnetworks in SNN are typically Convolutional Neural Networks (CNNs) when dealing with image data, or Recurrent Neural Networks (RNNs) for sequential data like time series or text. Training SNNs involves creating pairs of similar and dissimilar inputs. Here, the network learns to minimize distance between similar pairs and maximize distance between different pairs. The likely metrics used for evaluation of SNNs are precision, recall, the Area Under ROC Curve (AUC), etc., ensuring robust performance on different healthcare tasks.

SNNs excel in tasks requiring precise comparison and matching, leading to improved diagnostic accuracy. These kinds of neural network can handle large datasets efficiently, making them suitable for big data applications in healthcare. In addition, SNNs can be applied to various data types, including images, text, and genomic sequences, demonstrating their broad applicability. SNN accuracy varies greatly depending on the quality and size of the training data. Bad data make bad comparisons. However, training and deploying SNNs requires enormous computational power, eliminating a large number of healthcare institutions. Implementation and fine-tuning of SNNs is not straightforward; it requires an expert in neural networks as well as domain-specific knowledge.

With respect to this DAT-based project, the above schemes can be especially informative by exploiting the SNN’s ability to compare pre- and post-therapy data in order to quantify efficacy before implementation thereof. By learning the features associated with the pharmacogenetic response, a network can recognize signatures of treatment efficacy and provide a basis for quantitatively measuring therapy outcomes. This strategy is consistent with the SNN advantages reported by Feng and Chaspari in [12], which include high precision and scalability, and simultaneously overcome the difficulty of producing reliable input data for evaluations.

State-of-the-art SNN architectures highlight the disruptive ability of Siamese Neural Networks in healthcare promising better patient matching, disease diagnosis, and genomic analysis solutions. Complementary to these advancements, despite the challenges such as data quality and high computational requirement in SNN, built-in benefits of more robust accuracy with respect to scalability provide it a unique place in modern healthcare. This article can help the readers to better understand and apply SNNs in scientific investigation, such as some novel projects of studying dolphin-based therapies on pediatrics with cerebral palsy, which ultimately contributes to more objective and effective therapeutic evaluations.

In a different context, Feng and Chaspari in [12] explore the use of Siamese Neural Networks for the recognition of continuous speech emotions in ambulatory settings. This application is particularly relevant for mobile health, allowing real-time monitoring of patients’ emotional states. Although this approach offers real-time processing capabilities, it is sensitive to noise and requires clean input data to function effectively. Yang et al. in [13] use Siamese Neural Networks to extract drug–drug interactions from biomedical text. This method automates the extraction process, significantly improving drug safety by identifying potential adverse interactions. The main advantage of this approach is its ability to handle large volumes of textual data, although its effectiveness is contingent upon the quality of the input text. Cha and Vaidya in [14] applied Siamese Neural Networks for the detection of anomalies in healthcare data, grouped anomalies that could indicate potential health problems. This method enhances data security and anomaly detection, but is computationally intensive and can produce false positives. Koch et al. in [15] focus on one-shot image recognition using Siamese Neural Networks. This application is beneficial for diagnosing rare diseases in which only a single example may be available. The key advantage is the minimal training data required, though the network’s performance can be limited by the quality of the single samples available.

Siamese Neural Networks are also used by Tajbakhsh et al. in [16] to automate image analysis in healthcare and hence improve the diagnostic accuracy through deep analysis of images being compared. This is a great method for extracting rich features from images, but also comes along with high computational costs and intricate preprocessing. For instance, Yu et al. in [17] investigate the use of Siamese networks for non-intrusive load monitoring (which is more related to facility management) and show the versatility and adaptation of a Siamese network on a variety of types of data. It has the advantage of being non-invasive but less directly relevant to patient care. In [18], Strittmatter et al. investigate SNN application in biomedical image registration that allows one to align images of multiple modalities. This latter property makes the application critical for multimodal analysis: a technique used to heighten image alignment and hence improve reliance in deeper ML applications, though it necessitates high-quality input images. Satapathy et al. in [19] demonstrate that SNN can be used for classification in biosignal processing, with relevance to a problem known as sleep stage scoring. It achieves the best accuracy for biosignal classification of this subset, with high-quality signal data and feature processing necessary overall to achieve success in sleep studies. Most notably, SNN is applied to diagnostic predictions with uncertainty estimation by Utkin et al. in [20], an indispensable process for evaluating how confident one should be of the diagnosis results. This method results in a more confident diagnosis but is computationally heavy and requires extensive training data.

The similarities among these studies lie in their use of SNN to compare data inputs for various healthcare applications, all aiming to improve diagnostic accuracy and provide more objective assessments. Differences include the types of data used (text, images, biosignals) and specific network architectures (convolutional, clustering-based). Advantages often include high accuracy and robustness, while disadvantages often involve high computational requirements and dependence on high-quality input data.

Relating these findings to this work, the comparative capabilities of SNN are particularly relevant. By comparing pre- and post-therapy data, these networks can identify biomarkers indicative of therapy effectiveness. The project’s focus on objective assessment aligns with the uncertainty estimation techniques discussed, enhancing the reliability of evaluations. Methodologies from studies on emotion recognition and anomaly detection can ensure robust assessments, making SNN a promising tool for evaluating Dolphin-Assisted Therapy’s impact on children with cerebral palsy.

## 3. Methodology

### 3.1. Electroencephalography

**Figure 2:**
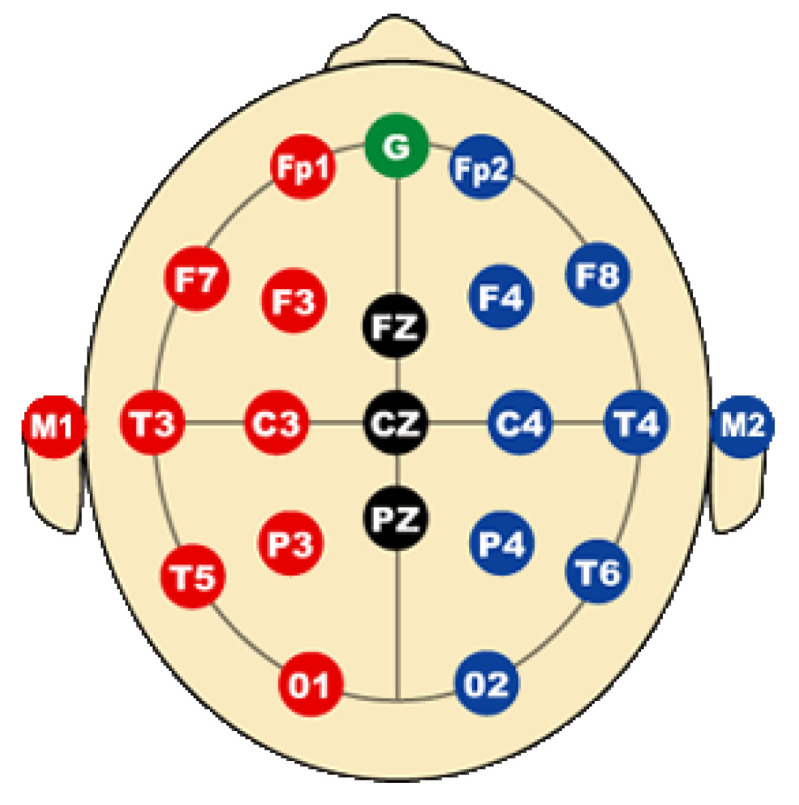
The International 10–20 system for the placement of electrodes in electroencephalography, The red electrodes indicate those placed on the left hemisphere of the brain, while the blue ones represent the right hemisphere. The black electrodes are central references, whereas the green electrode points to the nasion point.

In order to explain electroencephalography, it is essential to break down each aspect of the process for both specialists and those less familiar with the field. First, preparation for both the subject and the equipment is crucial. The participants are four children with SCP, the selection was based on their specific condition, such as age and health status. Before the procedure, participants must give their informed consent, ensuring that they or their parents understand the process. The scalp and forehead of the participant are then cleaned to reduce impedance and ensure good electrical contact with the electrodes. The electrodes are placed on the scalp according to the international 10–20 system, ensuring a precise placement. According to Figure 2, we place the electrode in the frontopolar area, i.e., $f_{p1}$ electrode.

**Figure 3:**
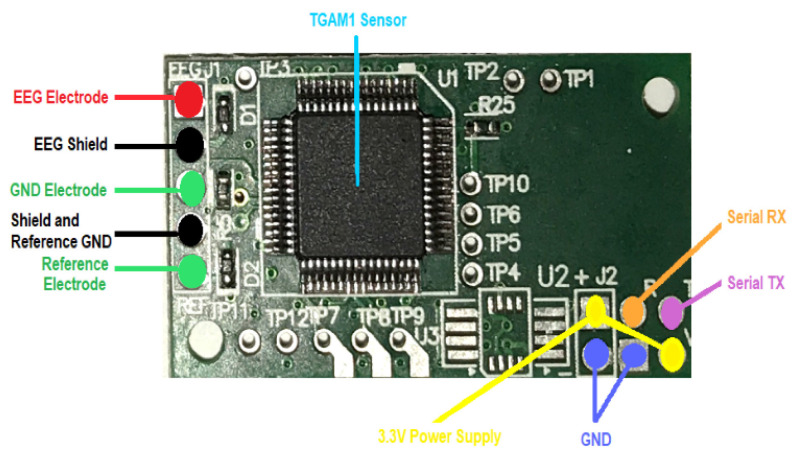
Electroencephalographic biosensor TGAM1 integrated with a communication with a serial module.

**Figure 4:**
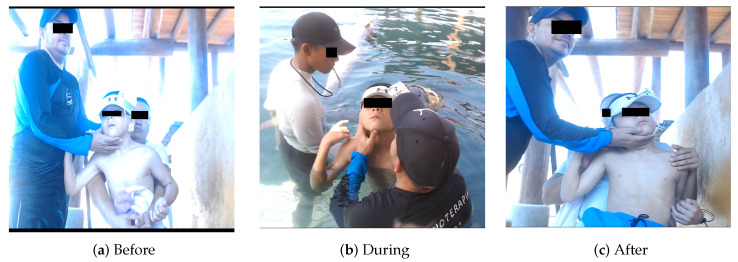
Procedure for acquiring EEG raw data Samples along a Dolphin-Assisted Therapy, (**a**) before-DAT stage, (**b**) during-DAT stage, (**c**) after-DAT stage.

The acquisition of EEG data starts with the correct setting of the equipment, such as the EEG amplifier and the acquisition software version 1.0. One such sensor, the TGAM1, is a sensor that is usually used to measure brainwave activity and can be found in products like NeuroSky’s MindWave [21]. Figure 3 shows the TGAM1 sensor integrated with communication with a serial module, which must be properly prepared to obtain accurate and reliable data. From Figure 4, observations were recorded before DAT for 60 s, during therapy for 5 min, and one minute after animal-assisted therapy. The recording is time-based according to the needs of the experiment. In this way, in Figure 3, each subfigure epitomizes a critical stage of the process. Figure 4a encapsulates the foundational Before phase, setting a clear precedent. Easily transitioning, Figure 4b unveils the dynamic During stage, brimming with real-time data capture and rapid transformations. Finally, Figure 4c shows the resolution found in the After phase, showcasing the culmination of precise data processing. This visual narrative not only illuminates the complexities inherent in EEG data acquisition but also underscores the unwavering dedication required at every step.

A comprehensive understanding of the four principal components of the TGAM1 sensor is imperative, and is delineated as follows:

1. Electroencephalogram Electrode (EE) is what captures the brainwave signal;
2. Reference Electrode (RE) helps to stabilize the signal and reduce noise;
3. Ground Electrode (GE) acts as a common baseline for the EEG recordings, reducing the impact of electrical noise and artifacts;
4. There is an amplifier along with the signal processor, which first acquires, then processes the electrical fluctuations in EE; these fluctuations are amplified and processed in order to obtain RAW signals [22].

The installation of the sensor has multiple steps. First, prepare the electrodes. Clean the ear pieces with an alcohol-moistened non-abrasive cloth, making sure they are free of dirt or oil, which can interfere with the signal. With this, it is ensured that the skin is completely dry, as even the smallest amount of moisture can affect the quality of the signal. The worn electrodes are checked as electrical elements can require replacement for accuracy. The EE is then plugged into the forehead or electrode $f_{p1}$ , and then the RE and GE electrodes are fixed in the back of the ears or in the earlobes. The headband or headset is made to fit snugly, but without discomfort, so contact with the skin is firm. Thus, the child with SCP is placed in front of the dolphin water tank, attempting to recreate a relaxed environment, in a comfortable chair with minimal movement and external distractions to improve the acquisition of stable readings. They should be settled and ask the child with SCP to try to remain as still as possible; even minimal facial movements can introduce artifacts into the EEG signal and reduce the quality of the results.

**Figure 5:**
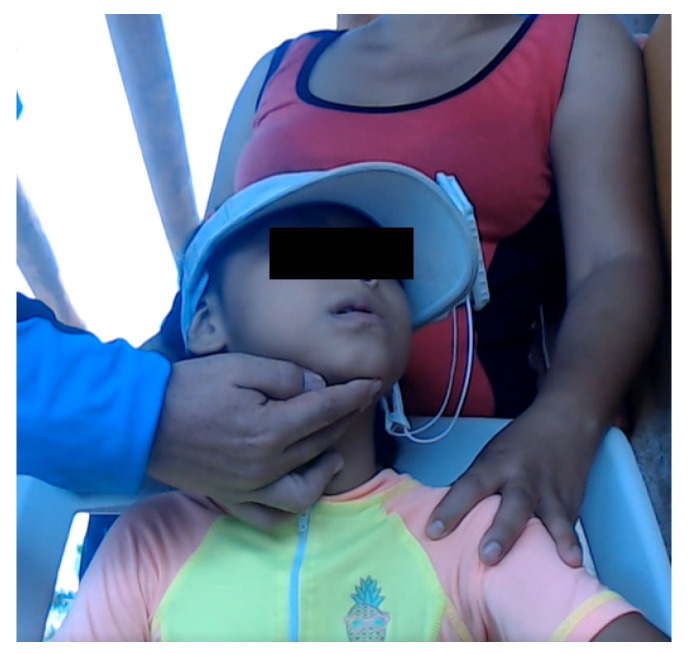
Placement of electrodes on the head of the child with spastic cerebral palsy, Electroencephalogram, reference, and ground, as well as the verification of the poor-signal flag.

This also needed to be followed by calibration and testing. We make sure the TGAM1 sensor is connected to the data-acquisition device; in this case, it is a computer with Matlab R2024a running. The sensor necessitates calibration; hence, an initial test reading involves recording 15 s of raw EEG time series data. This was procured from an SCP child at rest for a brief period, allowing the sensor to fully stabilize. Stabilization is determined when the internal poor-signal flag registers a value of less than or equal to 51 [23]. Once the signal quality is adequate, begin serving the data. Regularly monitor the signal quality and adjust the electrode placement or subject’s posture as necessary. It is important to maintain consistent recording conditions, as any external variables can potentially impact the data. The data are transmitted and downloaded as needed and with a periodicity of 512 samples per second, evaluating the signal quality by means of poor-signal indicator. If significant changes or drifts in the signal are detected, a recalibration is required. However, it can be certain that the TGAM1 sensor is properly trained for a correct working process to collect EEG data quite precisely and reliably, Figure 5.

After acquiring data, filters are employed in both pre-processing and post-processing stages to eliminate unwanted noise and artifacts. These filters include low-pass, high-pass, and notch filters, which help remove interference from sources such as electrical lines. The EEG signal is divided into time segments or epochs centered around specific events, such as stimuli in evoked potential studies, in this case the effect of DAT. Additionally, both physiological artifacts (such as blinks and muscle movements) and nonphysiological artifacts (like electrical interference) are identified and managed. There are different ways of analyzing EEG data. For this work, we analyzed the acquired data in two ways:

1. Event-Related Potentials (ERPs) are averages of neural responses that are time-locked to some event [24].
2. In the frequency domain, Fast Fourier Transform (FFT) breaks the signal down into its frequency components and exposes patterns of brain rhythms such as α , β , θ , γ , and δ waves [25].

It is imperative to interpret and visualize the data to derive meaningful insights from the results. Topographical maps have the advantage of seeing how electrical activity is distributed on the scalp, and spectrum power graphs allow comparing differences in energy distribution for different areas within frequency bands along with other popular representation methods. A network type, connectivity networks, are used to reveal functional dependencies between different parts of the brain. In addition, it is essential to be able to validate and replicate the results. The findings are statistically analyzed to ensure it is not a chance; thus, the experiments are conducted again to reassure the results. In the end, we need to protect against artifacts and noise which can threaten EEG data integrity, maintain high level of data protection and participant confidentiality, and acknowledge the physical limitations faced by EEG especially in terms of its lower spatial resolution as compared with other imaging techniques. These measures are essential for guaranteeing the robustness and validity of the acquired data, and they encapsulate the comprehensive process of executing this EEG study.

### 3.2. Mathematical Basis

Conventional methodologies encompass three critical processes: feature extraction, distance computation, and the retrieval of analogous features corresponding to a given query image or time series. These traditional systems face three principal challenges: (i) the semantic gap, (ii) the computation of similarity and dissimilarity, and (iii) the memory overhead required for storing visual descriptors. In this study, we advocate for the utilization of Siamese and triplet CNN architectures for the retrieval of both analogous and non-analogous EEG. The Siamese architecture employs two identical deep learning Convolutional Neural Networks with shared synaptic weights, using the contrastive cost function. The triplet architecture, akin to the Siamese architecture, employs three deep learning Convolutional Neural Networks with shared synaptic weights, but utilizes the triplet cost function.

In contrast to the manual process of content description employing low-level features such as frequency, these Convolutional Neural Networks (CNNs) provide a superior description by leveraging the intrinsic information of the electroencephalogram (EEG). This proposed methodology thereby mitigates the semantic gap. Distance calculation, embedded within the cost function of these neural architectures, facilitates learning of similarities and dissimilarities between various classes of EEGs in the database, thus enhancing discriminative power. Furthermore, it is unnecessary to store characteristic vectors, as the activation calculation of the neurons (forward propagation) can determine which time series most closely resemble the given EEG.

**Figure 6:**
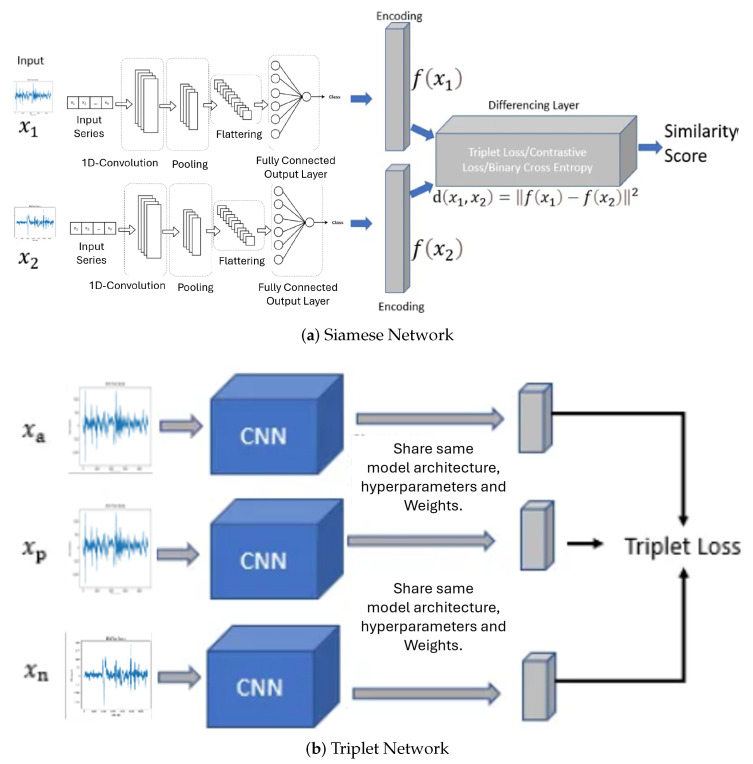
Architectures of the Siamese and triplet Convolutional Neural Networks.

Figure 6a,b depict the Siamese and triplet CNN architectures, respectively. Furthermore, Figure 6a illustrates that each network receives a pair of images as input, with the label variable indicating if the image pair is positive or negative.

The depicted Siamese Neural Network architecture comprises a pair of images as input to a system constituted by two identical subnets with shared synaptic weights. The output of these subnetworks is subsequently processed by a cost function. This cost function is designed to minimize a distance metric ( $L_{1}$ , $L_{2}$ , or cosine) between the feature representations of a positive pair of, e.g., ( $f\left(A\right)$ ) and ( $f\left(B\right)$ ), while maximizing this metric for a negative pair. In contrast to conventional CNNs, SNNs employ a contrastive cost function, which is defined by Equation (1) as follows:

(1) $$L=\frac{\left(1-Y\right){\left(D\right)}^{2}}{2}+\frac{\left(Y\right)\left(\max\left(0,m-D\right)\right)}{2}$$

In this context, let *D* represent the distance computed between the outputs of two Convolutional Neural Networks, f(A) and f(B) , which is defined as $D=\sqrt{{\left(G\left(X_{1}-X_{2}\right)\right)}^{2}}$ . The variable *Y* functions as a binary label, indicating whether the image pairs belong to the same class ( Y=1 ) or to different classes ( Y=0 ). Furthermore, *m* denotes a margin that specifies the required degree of similarity for time series within the same class, with m>0 . The presence of a margin implies that dissimilar pairs exceeding this threshold do not contribute to the loss.

The $L_{2}$ distance between the outputs of the triplet Siamese network can be expressed as follows:

(2) $$D_{L2}=\sqrt{\sum_{i=1}^{n}{\left(f{\left(A\right)}_{i}-f{\left(B\right)}_{i}\right)}^{2}}$$

where $f{\left(A\right)}_{i}$ and $f{\left(B\right)}_{i}$ signify the representations of the features of the electroencephalograms (EEGs) *A* and *B* obtained from the Siamese network, with *n* denoting the dimensionality of the feature space.

In accordance with Figure 6b, within the framework of a Siamese triplet network, the inputs consist of three distinct time series: (i) an anchor EEG denoted as $x_{a}$ , (ii) a positive EEG denoted as $x_{p}$ , and (iii) a negative EEG denoted as $x_{n}$ . The network subsequently computes the embedded representations of these time series, designated as $\mathrm{Net}(x_{a})$ , $\mathrm{Net}(x_{p})$ , and $\mathrm{Net}(x_{n})$ . Using the $L_{2}$ distance, the network produces two values: (i) the Euclidean distance between the anchor and the positive EEG, denoted as $D_{\mathrm{anchor}\text{-}\mathrm{positive}}={∥\mathrm{Net}\left(x_{a}\right)-\mathrm{Net}\left(x_{p}\right)∥}_{2}$ ; and (ii) the Euclidean distance between the anchor and the negative EEG, denoted as $D_{\mathrm{anchor}\text{-}\mathrm{negative}}={∥\mathrm{Net}\left(x_{a}\right)-\mathrm{Net}\left(x_{p}\right)∥}_{2}$ . Consequently, within the framework of the proposed triplet Siamese network, which utilizes inputs $x_{a}$ , $x_{p}$ , and $x_{n}$ along with their corresponding embedded representations Net(x) , the two distance metrics of the Euclidean norm ( $L_{2}$ ) between the respective feature vectors are formally defined as follows:

(3) $$D_{\mathrm{anchor}\text{-}\mathrm{positive}}={∥\mathrm{Net}\left(x_{a}\right)-\mathrm{Net}\left(x_{p}\right)∥}_{2}=\sqrt{\sum_{i=1}^{n}{\left(\mathrm{Net}{\left(x_{a}\right)}_{i}-\mathrm{Net}{\left(x_{p}\right)}_{i}\right)}^{2}}$$

(4) $$D_{\mathrm{anchor}\text{-}\mathrm{negative}}={∥\mathrm{Net}\left(x_{a}\right)-\mathrm{Net}\left(x_{n}\right)∥}_{2}=\sqrt{\sum_{i=1}^{n}{\left(\mathrm{Net}{\left(x_{a}\right)}_{i}-\mathrm{Net}{\left(x_{n}\right)}_{i}\right)}^{2}}$$

where $\mathrm{Net}{\left(x_{a}\right)}_{i}$ , $\mathrm{Net}{\left(x_{p}\right)}_{i}$ , and $\mathrm{Net}{\left(x_{n}\right)}_{i}$ are the feature representations of the images $x_{a}$ (anchor), $x_{p}$ (positive), and $x_{n}$ (negative) from the Siamese network, and *n* is the number of dimensions in the feature space, i.e., *x* and $x_{p}$ are images belonging to the same class, while $x_{n}$ belongs to a different class. In other words, triplet networks encode the distances of the images $x_{p}$ and $x_{n}$ with respect to the reference image *x*. It should be noted that the objective of triplet networks (similar to SNN) is to obtain a very large distance $∥\mathrm{Net}\left(x\right)-\mathrm{Net}\left(x^{n}\right){∥}_{2}$ between images *x* and $x^{n}$ , and a very short distance $∥\mathrm{Net}\left(x\right)-\mathrm{Net}\left(x^{p}\right){∥}_{2}$ between the images *x* and $x^{p}$ . The structure of this type of network can be seen in Figure 6b. For training, we used a triplet loss function, which is defined by:

(5) $$L=\sum_{i=1}^{N}{\left[∥\mathrm{Net}\left(x_{i}^{a}\right)-\mathrm{Net}\left(x_{i}^{p}\right){∥}_{2}^{2}-{∥\mathrm{Net}\left(x_{i}^{a}\right)-\mathrm{Net}\left(x_{i}^{n}\right)∥}_{2}^{2}+\alpha \right]}_{+}$$

where $x_{i}^{a}$ represents the anchor image, $x_{i}^{p}$ denotes the positive image (belonging to the same class as the anchor), $x_{i}^{n}$ signifies the negative image (belonging to a different class from the anchor), and α constitutes the margin imposed between positive and negative pairs. The operator ${\left[z\right]}_{+}$ signifies the hinge function, which yields the maximum of *z* and 0. Here, the function is described using the ${∥\cdot ∥}_{2}$ notation to denote the $L_{2}$ norm (Euclidean distance), which is crucial to ensure the desired distances between the anchor time series, positive and negative. Stated differently, these diverse architectures possess the capability to discern the inter-class and intra-class distances among images, addressing a prominent challenge currently recognized in the scholarly literature.

### 3.3. Proposed Architecture

**Figure 7:**
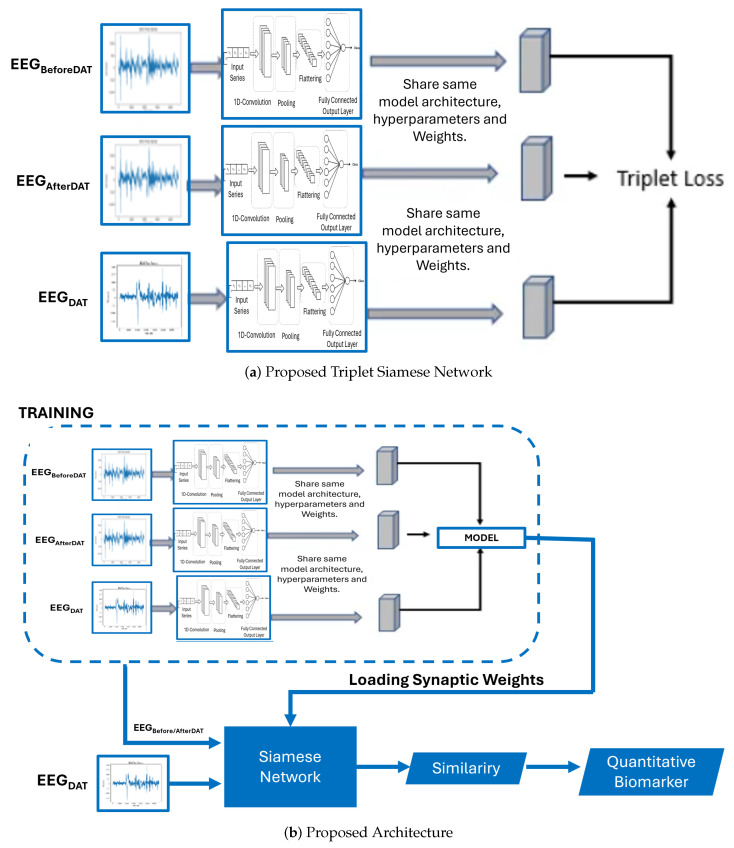
Proposed architectures of the Siamese and triplet Convolutional Neural Networks for assessing a quantitative biomarker.

The architecture of the Proposed Triplet Siamese Network for Time Series is delineated in Figure 7. It is pertinent to underscore that triplet Siamese networks are an extension of SNNs specifically engineered to discern and differentiate representations between pairs of electroencephalograms. In the context of time series analysis, the objective is to identify analogous or anomalous patterns within temporal sequences. Each time series is encapsulated as a sequence of characteristic vectors. The architecture employs two congruent branches of the Siamese network, with each branch processing an individual input time series. The shared layers are responsible for extracting salient features from the time series. The loss function is meticulously crafted to minimize the distance between the representations of analogous time series while maximizing the distance between the representations of dissimilar time series. The loss function is comprised of three components:

- Anchor term: Representation of the anchor time series.
- Positive term: Representation of a similar time series.
- Negative term: Representation of a dissimilar time series.

If two time series are similar, we want their representations to be close in the latent space. If two time series are dissimilar, we want their representations to be separated in the latent space. The principal hypothesis posits that the electroencephalographic patterns observed pre- and post-intervention exhibit analogous characteristics. However, should a high degree of similarity be detected during the DAT, it would imply that the DAT did not exert a substantial impact on children with SCP.

The proposed architecture harnesses the capabilities of these diverse architectures to mitigate the semantic gap and improve the discriminative efficacy of EEG. The proposed framework is depicted in Figure 6, which employs a triplet Siamese network and consists of two stages. The initial stage (dotted line block) represents the training phase, in which a triplet Siamese CNN is used, illustrated in Figure 6a. The EEG descriptors within the database are not retained; rather, only the synaptic weights of the trained network are preserved. The second stage is the process of retrieving similar EEG signals, in which one input of the SNN is the given query image, while the other input consists of all the time series in the database before or after DAT, introduced one by one. The level of similarity between each pair of EEGs is calculated in Equation (6) by applying the Euclidean distance to the two outputs of the Siamese Network:

(6) $$Similariry={∥\mathrm{Net}\left(EEG_{Before/AfterDAT}\right)-\mathrm{Net}\left(EEG_{DAT}\right)∥}_{2}$$

where $\mathrm{Net}(EEG_{Before/AfterDAT})$ and $\mathrm{Net}(EEG_{DAT})$ are the vectors of characteristics in the database of electroencephalograms before and after Dolphin-Assisted Therapy and EEGs during DAT, respectively, and ${∥\cdot ∥}_{2}$ is the $L_{2}$ norm. Once the similarity value between each pair $EEG_{Before/AfterDAT}↔EEG_{DAT}$ is obtained, the most similar ones to the given query are extracted. In this way, this system scheme is simplified as there is no need to store the descriptors. Additionally, the feature extractor and the similarity measure are embedded in a single process, a Siamese Neural Network.

### 3.4. Quantitative Biomarker

Then, we used a triplet network, whose training is performed as detailed in Figure 6b. In this scheme, a binary label is not necessary; instead, we introduce three images into the network, two of the same class, and one image of a different class. The training is responsible for learning that images with the same semantic content should have very close distances, and images with different semantic content should have a very large distance. For the query stage, we only use two networks (instead of three) since ultimately all three networks share the same synaptic weights. As a result, we will have a Siamese network that will obtain the similarity level between the query image and each of the images in the database. As in the Siamese architecture, the image feature vectors are not stored and the similarity calculation, given by Equation (6), they are performed within the same neural architecture, as seen in Figure 7. Once the similarity between each pair of time series is obtained, the most similar EEG signals to the given query EEG signals are retrieved, when this time series is obtained during the DAT.

Finally, for the new quantitative biomarker proposed called $QB_{DAT}$ whose units are decibels (dB), we propose Equation (7), where it is observed that the more similarity there is between brain activity at rest ( $\mathrm{Net}(EEG_{Before/AfterDAT})$ ) compared to brain activity during Dolphin-Assisted Therapy ( $\mathrm{Net}(EEG_{DAT})$ ), the more $QB_{DAT}\to 0$ tends to zero. However, the more dissimilar it is, the more effective the therapy was for the patient, then $QB_{DAT}\to \infty$ .

(7) $$QB_{DAT}=10\log_{10}\frac{1}{{∥\mathrm{Net}\left(EEG_{Before/AfterDAT}\right)-\mathrm{Net}\left(EEG_{DAT}\right)∥}_{2}}\left(dB\right)$$

## 4. Experiments and Results

### 4.1. Experimental Setup

The experimental protocol involved the acquisition of EEG signals from four children diagnosed with cerebral palsy, all of whom underwent Dolphin-Assisted Therapy (DAT). It is important to mention that for all four subjects, the data were recorded on the frontopolar electrode in the forehead ( $fp_{1}$ ) using the TGAM1 module. The entire system was then segmented into three primary components.

1. A female bottlenose dolphin;
2. Intervention patients, specifically children with cerebral palsy;
3. EEG device, sensor TGAM1, Figure 3.

The interaction among the three subsystems is crucial in determining the efficacy of DAT in patients with cerebral palsy. In our analysis of EEG data, we decomposed the EEG signal into distinct functional frequency bands, δ (0.5–4 Hz), θ (4–8 Hz), α (8–12 Hz), β (12–30 Hz), γ (30–60 Hz), and all bands (0.5–60 Hz), by employing the Fast Fourier Transform (FFT) to estimate the Power Spectral Density (PSD) expressed in μ V/Hz [26]. Subsequently, we investigated the behavior of the three patients with cerebral palsy using FFT on EEG signals [27]. The RAW EEG data, which are time series representing cerebral brain activity (voltage versus time), were collected at three distinct intervals: (i) At rest or Before DAT or $EEG_{BeforeDAT}$ , Figure 4a, (ii) During DAT or $EEG_{DAT}$ , Figure 4b, and (iii) Figure 4c or $EEG_{AfterDAT}$ , Figure 4c. All time series of EEG signals, regardless of whether they were captured, were recorded using the first frontopolar electrode ( $f_{p1}$ ) with a TGAM1 EEG biosensor module, as shown in Figure 3 [28].

The methodology used in this research was approved by the Ethics Committee of the National Polytechnic Institute of Mexico, as indicated in the confidentiality commitment letter D/1477/2020. This document validates the procedures for sample collection and treatment given to bottlenose dolphins (*Tursiops truncatus*) by the research team. In addition, this project ensured the ethical use of patient data, with participants fully informed and giving consent for the use of data collected during the experiments. Before sample collection and device attachment, participants were informed about the entire process; those who did not consent were allowed to withdraw immediately. Participants who agreed to the methodology and materials for sample collection signed a written informed consent form on 25 January 2020. In addition, participants were informed about the tests that would be performed on them, although some did not fully understand the details, before entering the tank with the cetaceans. The winter season was chosen to ensure that the temperature at noon during testing did not exceed 30 °C, while the measuring equipment was kept at temperatures not exceeding 20 °C. After sample collection, the adequacy of the data was evaluated. If found to be insufficient, participants were asked to remain in the tank for an additional sample collection, not exceeding 5 min.

### 4.2. Exploratory Data Analysis

Exploratory Data Analysis (EDA) forms a crucial part of the data-analysis pipeline in this study in that representations and visualizations of the main features in our dataset are for the most part displayed using graphical methods. Thus, the main aim of EDA is to reveal correlations, test assumptions and detect outliers by means of statistical graphics and other data-visualization methods. This, in turn, enables EDA and helps in comprehending the structure of the dataset of the electroencephalographic data, detects outliers and trends, and reduces the insight which helps further analyses and the decision-making.

**Figure 8:**
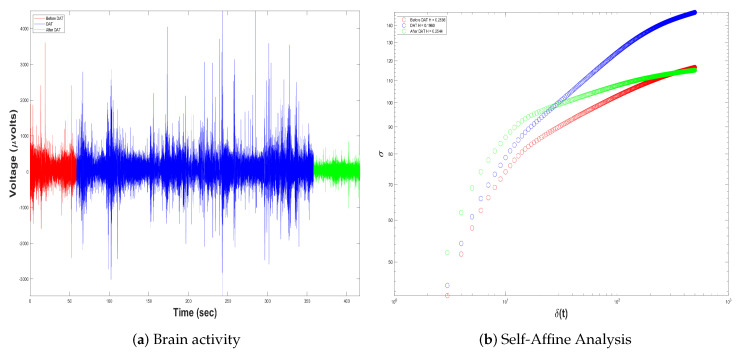
Event-related potentials before (in red), during (in blue), and after (in green) Dolphin-Assisted Therapy. (**a**) Raw brain activity in μ V, and (**b**) Self-Affine Analysis of signals in (**a**).

**Figure 9:**
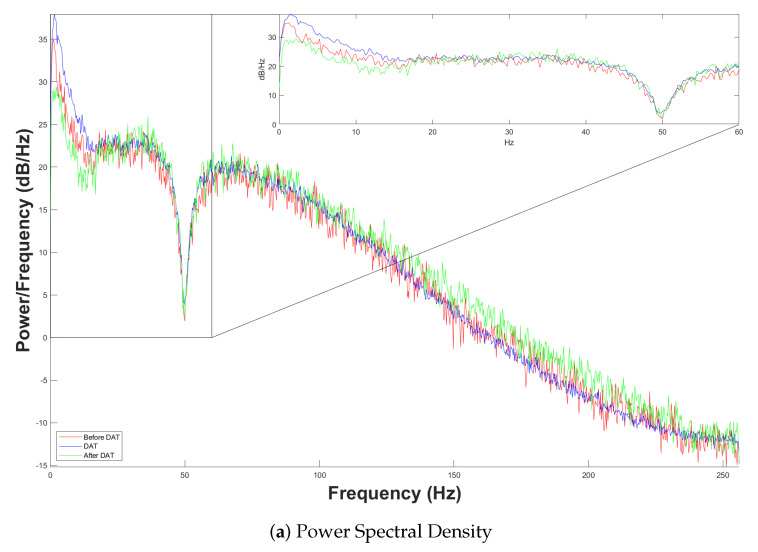

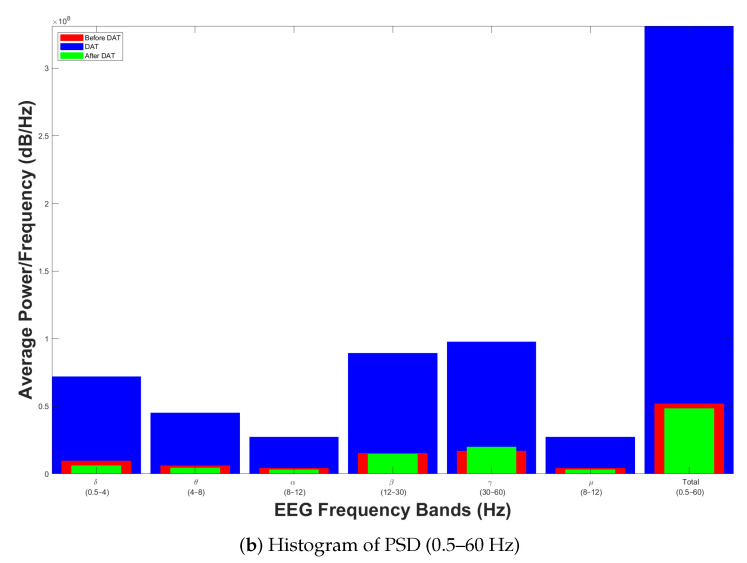
Power spectral density. (**a**) PSD from 0 to 256 Hz, and (**b**) histogram of fundamental brain rhythms.

In this way, remembering that Equation (7) defines the mathematical formula of the proposed quantitative biomarker in the form of decibels, it measures the deviation from the worst-case scenario. More specifically, it measures the similarity in signal behavior during the DAT and at rest (pre-/post-DAT). We provisionally identify the similarity in two different signal-analysis methods; the basic idea we want to explore is as follows:

1. ERP are averages around neural responses of the child with SCP during Dolphin-Assisted Therapy; in this case, two events are measured: (i) brain activity during DAT and (ii) brain activity before and after DAT. Hence, we propose to first look into the self-affine properties of these fluctuations in isolation by performing a Self-Affine Analysis (SAA) on the activity at these three points. Figure 8 indicates the cerebral activity throughout the ERP over the three EEG signal-acquisition epochs. Red denotes before the DAT, blue during DAT, and green after the DAT.
  The graph presented in Figure 8b illustrates the SAA. It appears at first glance that we have proven our hypothesis based upon the closeness or similarity of the curves immediately before and after the DAT. However, this view is myopic and reductive. Nonetheless, this interpretation is both biased and overly simplistic. Therefore, we will delineate the distinctions between the inflection points of each curve, namely to find the crossover. The crossover of the signal is the point where the signal possess a particular reference line or threshold, frequently the zero line, through a graph. It is important because this represents a change in the phase and/or direction that will be critical in a fractal process. Knowing when these crossovers occur allows us to better analyze how the brain signal behaves and makes proper adjustments to optimize performance. It is also discernible in Figure 8b that the crossover of the EEG signal of a child with SCP before DAT is quantified as CB=11 , after DAT it is CA=17 , and during DAT it is CD=107 . The similarity metric adopted here is the absolute distance between the curves before and after DAT or SBA , as defined by Equation (8). In contrast, the similarity of the curve during DAT or SDR was quantified as the mean distance to the pre- and post-DAT curves, as articulated in Equation (9).
(8) $$\begin{array}{ccc} SBA & = & \left|CA-CB\right| \end{array}$$
(9) $$\begin{array}{ccc} SDR & = & \frac{\left|CA-CD\right|+\left|CD-CB\right|}{2} \end{array}$$
  Thus, the directional shift seen in the curves of Figure 8b at SBA=6 and SDR=93 can be explained by the inclusion of the DAT. Then, the main finding was a larger similarity between the child’s activity and the SCP at rest and we also found interesting differences in the significance of these deviations for Dolphin-Assisted Therapy.
2. In the frequency domain, we use Fast Fourier Transform in order to break the brain activity down into its main frequency components and to filter patterns of brain rhythms such as δ , θ , α , β , and γ waves, i.e., the entire frequency spectrum ranging from 0.5 Hz to 60 Hz, systematically partitioned into five distinct sub-bands. Precisely, we use the Welch power spectrum, which is a more advanced method for estimating the Power Spectral Density (PSD) of a electroencephalographic signal. This technique is particularly valued for its ability to analyze this biosignal in terms of frequency content very effectively, even if there is noise. This approach of Welch divides the signal into overlapping segments, windows each segment using some window function, and then computes the Fourier transform of each segment. These are then averaged together to provide a more low-noise, and more accurate, estimate of the PSD. The main benefits of the Welch power spectrum are the ability to reduce the noise variance and the ability to reduce the noise variance nonstationary signals. This has a stabilizing effect; by averaging periodograms over overlapping segments, noise and fluctuations are suppressed and a more interpretable spectral estimate is obtained.
  Figure 9a illustrates the power spectral density of neural activity within the frequency range of 0 to 256 Hz. This observation is attributable to the TGAM1 sensor’s sampling rate of 512 samples per second (sps). In accordance with the Nyquist theorem, as delineated by Equation (10), the following relationship holds:
(10) $$f_{s}\geq 2f_{\max}$$
  where $f_{s}$ denotes the sampling frequency and $f_{\max}$ represents the maximum frequency contained within the signal. This equation asserts that the sampling frequency must be no less than twice the maximum frequency present in the signal to prevent aliasing. Also, Figure 9a shows that in the range of interest, it fluctuates between 0.5 Hz to $f_{\max}=60$ Hz. After $f_{\max}$ , a constant attenuation begins up to 256 Hz. This is because the human brain does not present activity above 60 Hz, so according to Equation (10), it would be sufficient with a $f_{s}=120$ sps, but the TGAM1 is programmed with $f_{s}=512$ sps. In addition, the Welch power spectrum is useful because it is able to effectively separate the frequency content of an EEG signal for its analysis and its interpretation into the following sub-bands: δ (0.5–4 Hz), θ (4–8 Hz), α (8–12 Hz), β (12–30 Hz), γ (30–60 Hz). Thus, Figure 9b shows the histogram of power spectrum density as a function of relative units for major brain rhythms. Interestingly, brain activity remains quite consistent at rest. The density of the child’s brain power spectrum from everything else (an outside stimulus during the DAT) hugely separates it from all other signals.

The need for Exploratory Data Analysis (EDA) is obvious given that neither PSD nor SAA have been applied previously to EEG signals processed from SCP children along with a Siamese Network. Thanks to the Welch PSD method, this can be accurately and consistently determined for the characteristic brainwave patterns related to cerebral palsy and their deviations. Finally, SAA also found fractal properties and self-correlation in the EEG signals which may provide scale-free traces of the electrical brain activity in patients with cerebral palsy. Overall, the combination of these methods provides a thorough analysis of EEG data that can identify small characteristics and trends associated with over states or conditions which might prove crucial for diagnostic purposes, treatment planning, and monitoring the effectiveness of Dolphin-Assisted Therapy.

### 4.3. Discussion

**Figure 10:**
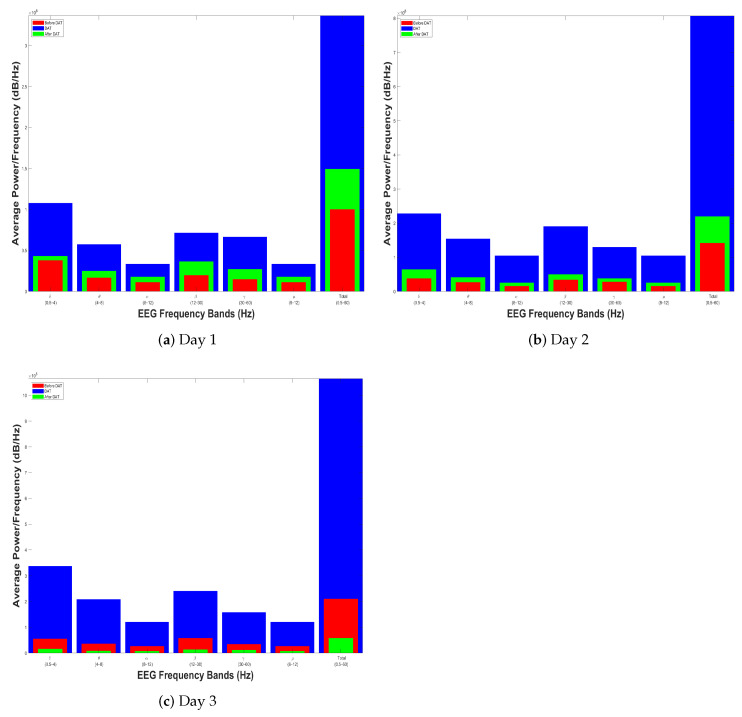
Power spectral density before (in red), during (in blue), and after (in green) the Dolphin-Assisted Therapy of Patient 1.

**Figure 11:**
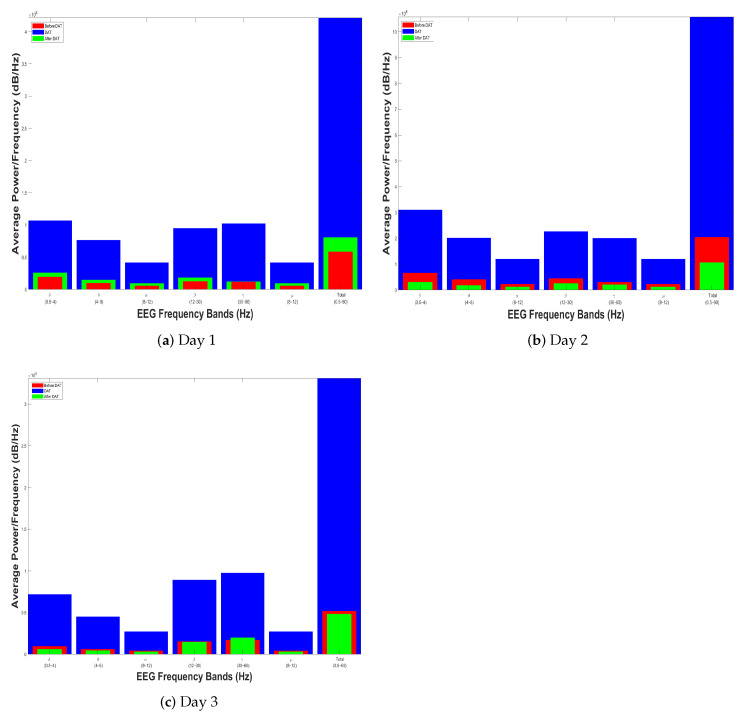
Power spectral density before (in red), during (in blue), and after (in green) the Dolphin-Assisted Therapy of Patient 2.

In Section 4.2, a single example is shown that initially demonstrates the similarity of the electroencephalographic signals. Thus, we display first the histograms of two out of four children with spastic cerebral palsy (henceforth referred to as patients) at the same age who took part in the experiment described in Section 4.1. Thus, Figure 10 and Figure 11, respectively, show the results of the power spectral density measurement of two patients. In all these cases, it can be observed that the signals are not equivalent in terms of normalized PSD during DAT compared to at rest, either before or after their therapy.

**Figure 12:**
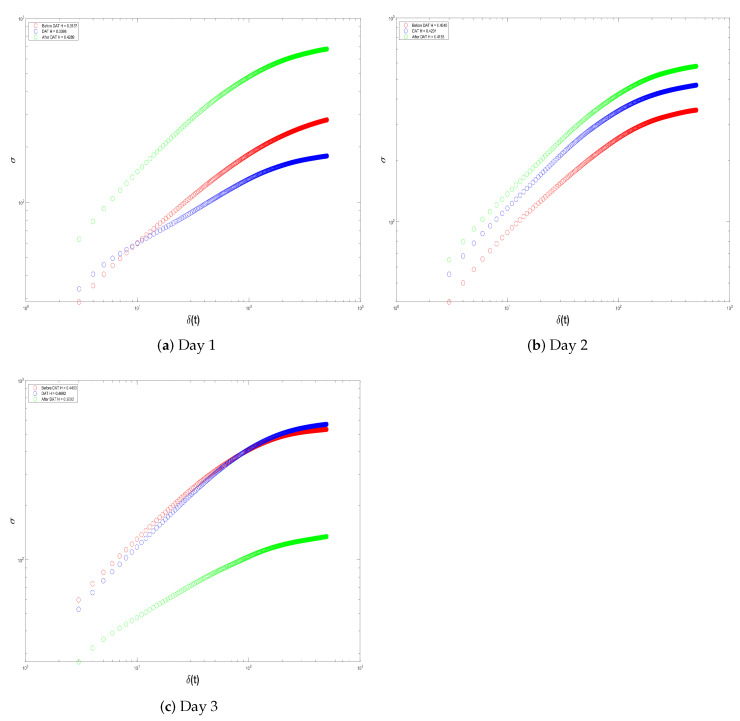
Self-Affine Analysis (in red), during (in blue), and after (in green) the Dolphin-Assisted Therapy of Patient 1.

**Figure 13:**
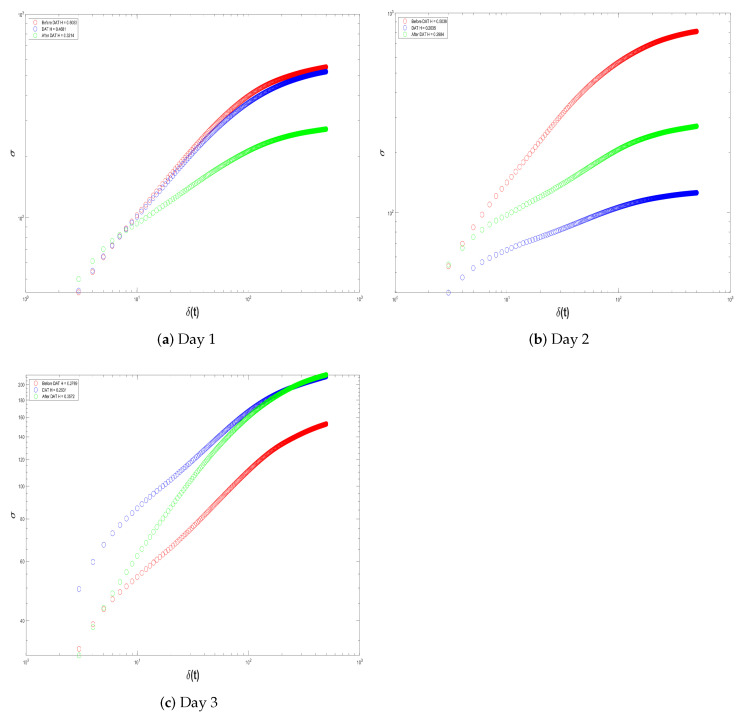
Self-Affine Analysis before (in red), during (in blue), and after (in green) the Dolphin-Assisted Therapy of Patient 2.

Figure 12 and Figure 13 show the Self-Affine Analysis of the samples presented in histograms depicted by Figure 10 and Figure 11. Here, the important thing is to know the differences between the inflection points. Thus, Table 1 shows the calculation of SBA and SDR , calculated from Equations (8) and (9). In all cases, at least for these two patients, it can be noted that the differences in the patient’s resting state are smaller than when they are subject to DAT.

On the one hand, Event-Related Potentials (ERPs), which represent averaged neural responses synchronized with specific events, offer profound insights into cerebral activity [24]. Furthermore, in the frequency domain, Fast Fourier Transform (FFT) dissects the EEG signal into its constituent frequency components, elucidating brain rhythms such as α , β , θ , γ , and δ waves [25]. Leveraging this information, we propose the utilization of SNNs to discern similarities in EEG signals of children with cerebral palsy. By examining EEG data pre- and post-Dolphin-Assisted Therapy, the Siamese network can proficiently compare these signals, capitalizing on the high degree of intrinsic similarity to enhance our comprehension of the therapy’s efficacy.

**Figure 14:**
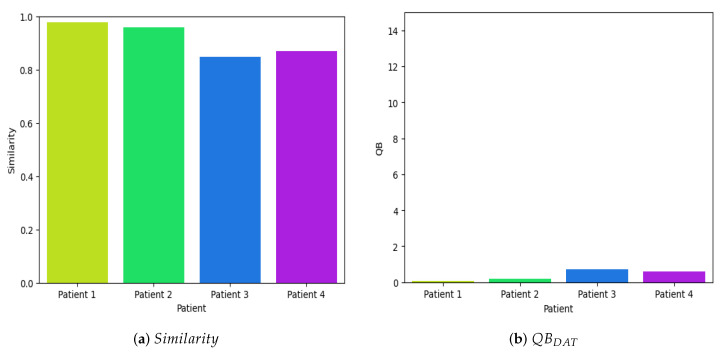
Quantitative evaluation of the efficiency of Dolphin-Assisted Therapy at rest, i.e., $EEG_{Before/AfterDAT}$ .

On the other hand, to evaluate the efficacy of the proposed model, four random samples were extracted from four children diagnosed with cerebral palsy. These samples exhibited an average similarity index of 0.9150, while the overall mean similarity across the entire database was 0.8753, $EEG_{Before/AfterDAT}$ . Figure 14a illustrates the similarity metrics of the samples in relation to the resting time series database, specifically those obtained before and after the children underwent Dolphin-Assisted Therapy. These findings are anticipated because the samples are inherently expected to exhibit a high degree of similarity to the database.

Using Equation (7), as depicted in Figure 14b, it can be discerned that the perturbations in the neuronal voltage exhibit minimal variance, rendering them statistically insignificant and approaching null values, since we obtain on average 0.3939 dB of efficiency. This observation does not imply the ineffectiveness of Dolphin-Assisted Therapy in activating specific cerebral regions; rather, the experiment is solely designed to evaluate the efficacy of the proposed model.

**Figure 15:**
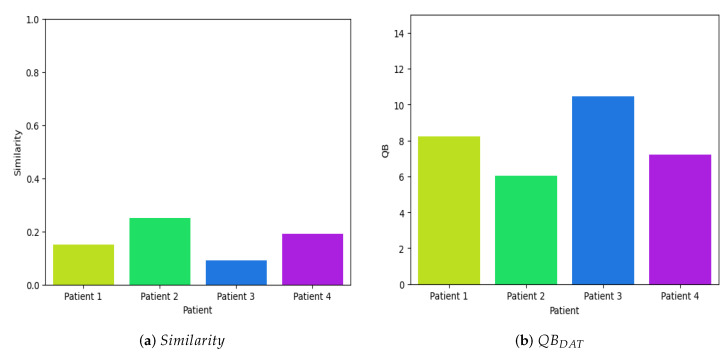
Quantitative evaluation of efficiency during a Dolphin-Assisted Therapy, i.e., $EEG_{DAT}$ .

In contrast, to evaluate the efficacy of Dolphin-Assisted Therapies, four random samples were obtained from four children diagnosed with cerebral palsy. These samples exhibited an average similarity index of 0.17, compared to a general mean similarity index of 0.2353 in all samples in the database $EEG_{DAT}$ . Figure 15a illustrates the similarity of these samples in relation to the time series database during Dolphin-Assisted Therapy sessions, specifically when the child is involved in this alternative therapeutic intervention. The observed results indicate a low similarity with the database, suggesting that Dolphin-Assisted Therapy induces distinct activity patterns compared to the typical activity of the patient.

The usage of SNNs in this study, which we analyzed in the present work despite the small sample size, is proof for their possible merit as biomarkers predicting efficacy of DAT treatment among children with SCP. The architecture of the SNN stands out in its capability to learn and generalize patterns of similarity between paired inputs, making it well adapted for processing small datasets. This property makes it ideally suitable for medical studies due to their often limited size of big data needs [15].

Evaluation in SCP Traditional assessment methods used within the context of a systematic review are often based on subjective outcomes that can be influenced by significant different measures between assessors. Our method exploits SNNs to yield a more objective and quantifiable assessment by analyzing pre- and post-DAT EEG signals. The obtained high average similarity index of 0.9150 in our study indicated that the network consistently recognized patterns correlating with therapeutic outcomes, despite a low sample size [29].

It is crucial to note that while our dataset is limited, the promising results obtained lay a strong foundation for further research. Future studies will benefit from larger datasets, allowing for more comprehensive training and validation of the SNN model. Additionally, incorporating longitudinal data can provide deeper insights into the sustained effects of DAT on SCP patients [30].

Moreover, the unique characteristics observed in neuronal-voltage shifts (with an average variance of 1.6987 dB between therapy and rest states) support SNN as being able to adequately represent significant brain activity modifications due to DAT. These results are in line with recent works that show high specificity of SNNs to be applied in clinical scenarios where data are scarce [31]. Finally, the uses of both ERP and FFT analyses supplement each other; together they create a more comprehensive framework for assessment [32].

Reapplying Equation (7) as depicted in Figure 15b reveals substantial disparities in neuronal voltage perturbations, with a mean value of these four samples at 7.9825 dB compared to 6.2838 dB for the entire dataset, supporting the assertion that Dolphin-Assisted Therapy selectively activates specific brain regions exclusively during this unconventional therapeutic intervention. Additionally, recent studies have shown that SNNs are well-suited for similarity-comparison tasks due to their unique architecture, which effectively extracts and compares features within the same feature space [15,33], making them critical for comparing pre- and post-therapy EEG signals to identify subtle changes indicative of therapeutic efficacy.

Previous comparative studies in other medical applications demonstrated that SNNs prevailed over classical methods with respect to accuracy and robustness. For example, SNNs are efficient in detecting progression over time with precision in tasks like medical image analysis [34,35], where longitudinal imaging data are essential. In terms of fine-grain comparative analysis, these result seem to prove rather effective SNN schemes.

In addition, recent developments in the domain of deep learning for EEG signal analysis also back our decision to use SNN. Other techniques such as Support Vector Machines (SVMs) and CNN models are also quite commonly used, yet typically these require a large pre-processing stage and may not account for the nuanced temporal interactions within EEG data in the same manner that SNN-based methods can [36,37]. Meanwhile, SNNs can use temporal dependencies and complex patterns of data; this is important for evaluating brain activity in children with SCP who undergo DAT.

The similarity index on average, with 0.9150, in our study is very high, which should be considered as an impressive enhancement over the baseline methods for extraction of therapeutic effects to reflect its advanced ability compared with previous ones. This means such a performance metric is relevant to our research objectives, and suggests that the base design of SNNs around pairwise comparison can potentially make it suitable for further investigation. Finally, the altered neuronal voltage fluctuations measured acutely following treatment demonstrate that SNN of forecasting may detect subtle but essential modifications in EEG patterns [38]. Namely, although other methods like SVM, RNN, and CNN schemes have their unique advantages over SNN architectures, the inherent structure of SNN for similarity comparison tasks provides an edge. The reliable and consistent performance as well as strong validation of SNNs in our application suggest that they have indeed the potential to be a robust, objective biomarker for efficacy assessment of DAT in pediatric SCP.

### 4.4. Comparison with Other State-of-the-Art Methods

In this section, the proposed SNN is to be used as a biomarker for data on sensitivity and specificity in pediatric clinical trials that evaluate the efficacy of DAT treatment.

Table 2 provides a comparative analysis of the proposed Siamese Neural Network (SNN) against other state-of-the-art methods such as Support Vector Machine (SVM) [39], Convolutional Neural Network (CNN) [40], Recurrent Neural Network (RNN) [41], K-Nearest Neighbors (KNN), and Random Forest (RF). The performance metric used for comparison is the similarity index, which measures how well each method can identify therapeutic responses in EEG data from children with Spastic Cerebral Palsy (SCP) undergoing Dolphin-Assisted Therapy (DAT).

The similarity index of our proposed SNN method was 0.9150, the highest among all versions compared and shown in Table 2. This shows that SNN can be a strong approach for measuring similarity and differences of pre-therapy case EEG patterns with post-therapy cases; hence, it is an effective tool to evaluate DAT. The high similarity index of therapeutic impacts indicates the power of SNNs to learn complex patterns in EEG data, allowing deeper insight into treatment effects than traditional approaches. The SVM method, which is a traditional machine learning algorithm, obtained a similarity index of 0.8753 compared to the use of transfer learning. Although SVMs are powerful and practical for exact data matching, the subtle high-frequency dynamics seen in EEG has this weakness [39]. The similarity index of CNNs, which are very much used in depth image-analysis tasks, was a little worse, 0.8600; CNNs are great, but not always accurate for detailed comparison work such as similarity [40]. Each method has its own strengths and weaknesses. The SNN’s strength lies in its ability to accurately compare pre- and post-therapy signals within the same feature space, providing a high degree of accuracy in detecting therapeutic responses. However, the primary limitation of the SNN approach in this study is the small size of the dataset, which could impact the generalizability of the results. Future research with larger datasets will be essential to validate these findings.

On the other hand, SVMs are known for their robustness and effectiveness in various applications, but their performance decreases with the complexity of temporal EEG data [39]. RNNs are designed to handle sequential data and capture temporal dependencies well, achieving a similarity index of 0.8700. However, they require extensive computational resources and can be challenging to train effectively [41]. In the same way, KNN and RF, while simpler and more intuitive methods, show lower similarity indices of 0.8450 and 0.8550, respectively. KNN is effective for small datasets, but struggles with high-dimensional data, while RF offers good accuracy and robustness, but can be computationally intensive when dealing with large datasets [42,43].

The high sensitivity of the approach for patients with SCP in this study demonstrates its potential to serve as a sensitive and readily observable biomarker response endpoint of DAT treatment efficacy and it should be utilized within clinical trials investigating pediatric DAT therapies. The results suggest that a less subjective measure of the effect on class members of therapeutic treatments is possible using SNN compared with traditional methods. The limitations in our study, particularly the low sample size, reflect that more longitudinal studies with larger cohort sizes are warranted to confirm these findings and the potential clinical utility of SNNs. The next steps in research should include expanding the data and including patient populations with a wider distribution to increase generalizability. In addition, the performance and robustness of these methods can be strengthened by finding a way that works with SNNs for other neural networks or even hybrid/full approach versions. In general, this work sets the foundation for new innovative data-driven tools that have the ability to provide a more precise quantitative evaluation of the outcomes of the therapeutic intervention to help the patient respond.

Performance is finally evaluated based on a similarity index, which concludes that the SNN obtained yields an accuracy of 0.9150—the best quality dataset among them all [25].This shows the precision with which it is capable of detecting therapeutic responses and makes it a promising tool for evaluating the efficiency of DAT. In contrast, the main constraint of SNN is that given only a few data points, further research with larger samples will be required to validate these results. This section also provides a comparison to other methods (SVM, CNN, KNN, and RF) and talks about the advantages and disadvantages of these approaches as well. In brief, SNN has the potential to be a sensitive and valid instrument to distinguish between controls and persons with DAT treated at baseline through 12 months of follow-up, although AD/HV comparisons without treatment should also be studied.

## 5. Conclusions

Spastic cerebral palsy (SCP) represents the most prevalent subtype of cerebral palsy, characterized by motor impairments attributed to early brain injury. Dolphin-Assisted Therapy (DAT) has emerged as a promising adjunctive therapeutic modality for pediatric SCP patients. This investigation examines the feasibility of employing a Siamese network as a biomarker to evaluate the efficacy of Dolphin-Assisted Therapy in children with cerebral palsy.

Both ERP and FFT can analyze the effect changes in brain activity or brain rhythms. This study implemented an SNN in EEG data from children diagnosed with cerebral palsy and compared their results before and after Dolphin-Assisted Therapy. The efficacy of the model was assessed with data from four children and demonstrated an average similarity index in new samples equal to 0.9150. High similarity metrics are observed before and after therapy in the results, which is to be expected given that methodologically these samples closely mimic those present within the database of origin.

The methodology encompasses the training of a simulation neural network, a specialized neural network architecture designed to assess input similarities, using data obtained from SCP patients participating in DAT sessions. The study results are encouraging, showing distinct patterns in the output of SNNs that correlate with symptomatic improvements in SCP after DAT. These findings underscore the potential applicability of SNN as an objective and quantifiable means of evaluating therapeutic interventions. Ultimately, it is clear that the variations in neuronal voltage fluctuations are considerable, leading to a notable deviation from zero. This deviation indicates a significant difference from the activity of the resting state. The average value of these four samples is 7.9825 dB, while the average value for the entire dataset is 6.2838 dB. Therefore, this supports the claim that Dolphin-Assisted Therapy specifically activates certain regions of the brain only during this unique therapeutic approach.

Dolphin-Assisted Therapy shows the largest differences in voltage perturbation at all neuronal groups by a factor of 7.9825 dB for four data samples, and there is an improvement to only 6.2838 dB for cleaning entire datasets, which may indicate that dolphin-augmented sounds activate separate brain regions in particular modes. SNNs are a variation for matching that work particularly well with similarity-comparison tasks, such as contrasting pre- and post-therapy EEG signals [15,33]. SNNs are more accurate and robust than traditional methods in medical image analysis, which can be observed from our comparative assessments of DAT studies [34,35]. Deep learning, which has achieved state-of-the-art results for many vision- and language-processing tasks captures fine-resolution temporal relationships in EEG data better compared to SVM or CNN schemes, as shown by advances on the front of deep learning with SNN [36]. The performance of our SNN was excellent, with a similarity index as high as 0.9150, showing that it can also detect therapeutic responses [38]. As for their merits, you could argue that SVMs are designed for exact matches, whereas RNN and CNN, with convolutional layers, might not be so adamant in feature dimensions being the same. The potential value of this effect as a stable peripheral biomarker to monitor DAT efficacy in children with cerebral palsy is supported by our SNN application.

It should be noted that although our dataset is small, with four patients, the very encouraging performance of generative score compression demonstrated here offers an excellent grounding for future research. In the future, larger datasets will become available that would enable an extensive training and validation of its model by SNNs. In addition, longitudinal data can also offer further understanding of the prolonged effect of DAT against SCP patients [30].

Finally, while the study sample size was small, our findings provide indicative evidence that SNN could be a useful biomarker for assessing DAT outcome in children with SCP. These results support further development and scale-up of this research, highlighting the necessity for objective data-driven tools related to therapeutic interventions.

In conclusion, this method shows promise as a sensitive and quantifiable biomarker of DAT-mediated efficacy. The results suggest that SNNs may provide a quantitative difference from standard methods. However, there were a number of limitations to the study that its authors acknowledged and further longitudinal studies with larger cohorts would be required to determine whether these findings are true or have implications for clinical work. Subsequent investigations are warranted to provide data of greater clinical significance in more diverse patient populations and for external validation purposes. SNN methods have been improved through the addition of traditional neural network techniques and/or hybrid approaches, which can be simply merged with these techniques to enhance their performance and robustness. Together, these data provide the basis for generating new machine learning-based methods that allow quantitative evaluation of therapeutic interventions and improved outcomes in patients.

## Figures and Tables

**Table 1:** Analysis of crossovers of Figure 12 and Figure 13, cells in gray represent *SBA*.

Patient	Day	Crossover			Similarity Based on Crossover	
		Before	During	After	*SBA*	*SDR*
1	1	210	193	203	**7**	13.5
	2	173	135	167	**6**	35
	3	116	170	149	**33**	37.5
2	1	146	187	132	**14**	48
	2	181	103	167	**14**	71
	3	156	151	159	**3**	6.5

**Table 2:** Comparison of proposed SNN with state-of-the-art methods.

Method	Key Features	Similarity Index	Strengths	Weaknesses
Proposed SNN	Siamese Neural Network trained on EEG data	0.9150	High accuracy in detecting therapeutic responses; Effective in comparing pre- and post-therapy signals	Limited dataset size
Support Vector Machine (SVM) [39]	Traditional machine learning approach	0.8753	Good for exact matches; Robust in many applications	Less effective with temporal relationships in EEG data
Convolutional Neural Network (CNN) [40]	Deep learning model with convolutional layers	0.8600	Excellent for image analysis; Can handle large datasets	Not as precise in similarity comparison tasks
Recurrent Neural Network (RNN) [41]	Deep learning model designed for sequential data	0.8700	Captures temporal dependencies well; Effective for sequential data	Requires extensive computational resources
K-Nearest Neighbors (KNN) [42]	Instance-based learning method	0.8450	Simple and intuitive; Effective for small datasets	Performance degrades with high-dimensional data
Random Forest (RF) [43]	Ensemble learning method using decision trees	0.8550	Good accuracy and interpretability; Robust to overfitting	Computationally intensive with large datasets

## Data Availability

The original contributions presented in the study are included in the article, further inquiries can be directed to the corresponding author.
