# Correction: Moreno Escobar et al. Employing Siamese Networks as Quantitative Biomarker for Assessing the Effect of Dolphin-Assisted Therapy on Pediatric Cerebral Palsy. *Brain Sci.* 2024, *14*, 778

**DOI:** 10.3390/brainsci15050425

**Published:** 2025-04-22

**Authors:** Jesús Jaime Moreno Escobar, Oswaldo Morales Matamoros, Erika Yolanda Aguilar del Villar, Hugo Quintana Espinosa, Liliana Chanona Hernández

**Affiliations:** 1Centro de Investigación en Computación, Instituto Politécnico Nacional, Ciudad de México 07700, Mexico; omoralesm@ipn.mx; 2Escuela Superior de Ingeniería Mecánica y Eléctrica, Unidad Zacatenco, Instituto Politécnico Nacional, Ciudad de México 07738, Mexico; eaguilard2000@alumno.ipn.mx (E.Y.A.d.V.); hquintana@ipn.mx (H.Q.E.); lchanona@gmail.com (L.C.H.)

## Error in Affiliation

An error was identified and corrected in the telephone extension originally listed in the original publication [1]. The initial entry showed the extension as 54970, while the correct number should be 56666.

## References

Errors in the References section were identified due to an improperly formatted BibTeX database. The content has been corrected as follows:

References [1,4–8,10–14,16–20,30–32,34,35,39–43] have been replaced with:

1.Himmelmann, K.; Uvebrant, P. Function and neuroimaging in cerebral palsy: A population-based study: Function and Neuroimaging in CP. *Dev. Med. Child Neurol.*
**2011**, *53*, 516–521. https://doi.org/10.1111/j.1469-8749.2011.03932.x.4.Ahmed, A.; Rosella, L.C.; Oskoui, M.; Watson, T.; Yang, S. Prevalence and temporal trends of cerebral palsy in children born from 2002 to 2017 in Ontario, Canada: Population-based cohort study. *Dev. Med. Child Neurol.*
**2022**, *65*, 243–253. https://doi.org/10.1111/dmcn.15324.5.Barron-Garza, F.; Coronado-Garza, M.; Gutierrez-Ramirez, S.; Ramos-Rincon, J.M.; Guzman-de la Garza, F.; Lozano-Morantes, A.; Flores-Rodriguez, A.; Nieto-Sanjuanero, A.; Alvarez-Villalobos, N.; Flores-Villarreal, M.; et al. Incidence of Cerebral Palsy, Risk Factors, and Neuroimaging in Northeast Mexico. *Pediatr. Neurol.*
**2023**, *143*, 50–58. https://doi.org/10.1016/j.pediatrneurol.2023.02.005.6.Pousada, M.; Guillamón, N.; Hernández-Encuentra, E.; Muñoz, E.; Redolar, D.; Boixadós, M.; Gómez-Zúñiga, B. Impact of Caring for a Child with Cerebral Palsy on the Quality of Life of Parents: A Systematic Review of the Literature. *J. Dev. Phys. Disabil.*
**2013**, *25*, 545–577. https://doi.org/10.1007/s10882-013-9332-6.7.Tohen-Zamudio, A.; Tohen-Bienvenu, A.; Aguilera-Zapata, J.M. La parálisis cerebral en México. *Salud Pública Méx.*
**2014**, *28*, 125–133. Available online: https://www.saludpublica.mx/index.php/spm/article/view/368 (accessed on 15 April 2025).8.Lerma-Castaño, P.R.; Chanaga-Gelves, M.V.; Llanos-Mosquera, J.M.; Castro-Serrato, L.E.; Romana-Cabrera, L. Virtual reality in gait rehabilitation in children with spastic cerebral palsy. *Rev. Mex. Neurocienc.*
**2022**, *23*, 1. https://doi.org/10.24875/rmn.21000001.10.Delgado, M.R. The Mexican Academy for Cerebral Palsy and Neurodevelopmental Disorders: New kid on the block. *Dev. Med. Child Neurol.*
**2016**, *58*, 109. https://doi.org/10.1111/dmcn.13008.11.Ibarra-Rodríguez, M.C. Navigating challenges with cerebral palsy in low- and middle-income countries. *Dev. Med. Child Neurol.*
**2024**, *67*, 278. https://doi.org/10.1111/dmcn.16195.12.Feng, K.; Chaspari, T. Few-Shot Learning in Emotion Recognition of Spontaneous Speech Using a Siamese Neural Network With Adaptive Sample Pair Formation. *IEEE Trans. Affect. Comput.*
**2023**, *14*, 1627–1633. https://doi.org/10.1109/TAFFC.2021.3109485.13.Yang, Z.; Tong, K.; Jin, S.; Wang, S.; Yang, C.; Jiang, F. CNN-Siam: Multimodal siamese CNN-based deep learning approach for drug–drug interaction prediction. *BMC Bioinform.*
**2023**, *24*, 1. https://doi.org/10.1186/s12859-023-05242-y.14.Cha, B.R.; Vaidya, B. Enhancing Human Activity Recognition with Siamese Networks: A Comparative Study of Contrastive and Triplet Learning Approaches. *Electronics*
**2024**, *13*, 1739. https://doi.org/10.3390/electronics13091739.16.Tajbakhsh, N.; Shin, J.Y.; Gurudu, S.R.; Hurst, R.T.; Kendall, C.B.; Gotway, M.B.; Liang, J. Convolutional Neural Net- works for Medical Image Analysis: Full Training or Fine Tuning? *IEEE Trans. Med. Imaging*
**2016**, *35*, 1299–1312. https://doi.org/10.1109/TMI.2016.2535302.17.Yu, M.; Wang, B.; Lu, L.; Bao, Z.; Qi, D. Non-Intrusive Adaptive Load Identification Based on Siamese Network. *IEEE Access*
**2022**, *10*, 11564–11573. https://doi.org/10.1109/ACCESS.2022.3145982.18.Strittmatter, A.; Schad, L.R.; Zöllner, F.G. Deep learning-based affine medical image registration for multimodal minimal- invasive image-guided interventions—A comparative study on generalizability. *Z. Med. Phys.*
**2024**, *34*, 291–317. https://doi.org/10.1016/j.zemedi.2023.05.003.19.Satapathy, S.K.; Brahma, B.; Panda, B.; Barsocchi, P.; Bhoi, A.K. Machine learning-empowered sleep staging classification using multi-modality signals. *BMC Med. Inform. Decis. Mak.*
**2024**, *24*, 1. https://doi.org/10.1186/s12911-024-02522-2.20.Utkin, L.; Kovalev, M.; Kasimov, E. Explanation of Siamese Neural Networks for Weakly Supervised Learning. *Comput. Inform.*
**2020**, *39*, 1172–1202. https://doi.org/10.31577/cai_2020_6_1172.30.Jafari, M.; Shoeibi, A.; Khodatars, M.; Bagherzadeh, S.; Shalbaf, A.; García, D.L.; Gorriz, J.M.; Acharya, U.R. Emotion recognition in EEG signals using deep learning methods: A review. *Comput. Biol. Med.*
**2023**, *165*, 107450. https://doi.org/10.1016/j.compbiomed.2023.107450.31.Alaverdyan, Z.; Jung, J.; Bouet, R.; Lartizien, C. Regularized siamese neural network for unsupervised outlier detection on brain multiparametric magnetic resonance imaging: Application to epilepsy lesion screening. *Med. Image Anal.*
**2020**, *60*, 101618. https://doi.org/10.1016/j.media.2019.101618.32.Dilts, R.; Trompisch, N.; Bergquist, T.M. Dolphin-Assisted Therapy for Children With Special Needs: A Pilot Study. *J. Creat. Ment. Health*
**2011**, *6*, 56–68. https://doi.org/10.1080/15401383.2011.557309.34.Zhang, Y.; Zhao, Y.; Huang, L.; Xia, L.; Tao, Q. Deep-learning-based groupwise registration for motion correction of cardiac *T*_1_ mapping. *arXiv*
**2024**. https://doi.org/10.48550/ARXIV.2406.12456.35.Litjens, G.; Kooi, T.; Bejnordi, B.E.; Setio, A.A.A.; Ciompi, F.; Ghafoorian, M.; van der Laak, J.A.W.M.; van Ginneken, B.; Sánchez, C.I. A survey on deep learning in medical image analysis. *Med. Image Anal.*
**2017**, *42*, 60–88.39.Kumari, S.; Khare, V.; Arora, P. Optimizing Seizure Detection: A Comparative Study of SVM, CNN, and RNN-LSTM. *Int. J. Comput. Methods Exp. Meas.*
**2024**, *12*, 405.40.Wang, X.; Ren, Y.; Luo, Z.; He, W.; Hong, J.; Huang, Y. Deep learning-based EEG emotion recognition: Current trends and future perspectives. *Front. Psychol.*
**2023**, *14*, 1126994.41.Shahtalebi, S.; Asif, A.; Mohammadi, A. Siamese Neural Networks for EEG-based Brain-computer Interfaces. In Proceedings of the 42nd Annual International Conference of the IEEE Engineering in Medicine & Biology Society (EMBC), Montreal, QC, Canada, 20–24 July 2020; pp. 442–446.42.Unnisa, Z.; Zia, S.; Butt, U.M.; Letchmunan, S.; Ilyas, S. Ensemble Usage for Classification of EEG Signals: A Review with Comparison. In *Augmented Cognition. Theoretical and Technological Approaches*; Springer: Cham, Switzerland, 2020; pp. 189–208.43.Ewen, J.B.; Levin, A.R. Neurobehavioral Biomarkers: An EEG Family Reunion. *J. Clin. Neurophysiol.*
**2021**, *39*, 129–134.

References [2,3,9,15,21,22,33,37] have been corrected as follows:

2.Chen, D.; Huang, M.; Yin, Y.; Gui, D.; Gu, Y.; Zhuang, T.; Chen, C.; Huo, K. Risk factors of cerebral palsy in children: A systematic review and meta-analysis. *Transl. Pediatr.*
**2022**, *11*, 4. https://doi.org/10.21037/tp-22-78.3.Lucas, B.R.; Elliott, E.J.; Coggan, S.; Pinto, R.Z.; Jirikowic, T.; McCoy, S.W.; Latimer, J. Interventions to improve gross motor performance in children with neurodevelopmental disorders: A meta-analysis. *BMC Pediatr.*
**2016**, *16*, 193. https://doi.org/10.1186/s12887-016-0731-6.9.Blumetti, F.C.; Belloti, J.C.; Tamaoki, M.J.S.; Pinto, J.A. Botulinum toxin type A in the treatment of lower limb spasticity in children with cerebral palsy. *Cochrane Database Syst. Rev.*
**2019**, *10*, 1–408. https://doi.org/10.1002/14651858.cd001408.pub2.15.Koch, G.R. Siamese Neural Networks for One-Shot Image Recognition. In Proceedings of the 2015 International Conference on Machine Learning, Lille, France, 7–9 July 2015. Available online: https://api.semanticscholar.org/CorpusID:13874643 (accessed on 15 April 2025).21.Wang, Y.X.; Lin, L.S.; Tsai, H.Y. EEG Analyzing Color Temperature Influence on Emotions Based on TGAM Module. In Proceedings of the 2023 IEEE 6th International Conference on Automation, Electronics and Electrical Engineering (AUTEEE), Shenyang, China, 15–17 December 2023; pp. 441–444. https://doi.org/10.1109/auteee60196.2023.10407397.22.Yu, F.; Yu, C.; Tian, Z.; Liu, X.; Cao, J.; Liu, L.; Du, C.; Jiang, M. Intelligent Wearable System With Motion and Emotion Recognition Based On Digital Twin Technology. *IEEE Internet Things J.*
**2024**, *11*, 26314–26328. https://doi.org/10.1109/JIOT.2024.3394244.33.Bertinetto, L.; Valmadre, J.; Henriques, J.F.; Vedaldi, A.; Torr, P.H.S. Fully-Convolutional Siamese Networks for Object Tracking. *arXiv*
**2016**. https://doi.org/10.48550/ARXIV.1606.09549.37.Bashivan, P.; Rish, I.; Yeasin, M.; Codella, N. Learning Representations from EEG with Deep Recurrent-Convolutional Neural Networks. *arXiv*
**2015**, arXiv:1511.06448.

Following the reference updates, the in-text citations of the modified references have been aligned with the correct references.

The authors state that the scientific conclusions are unaffected. This correction was approved by the Academic Editor. The original publication has also been updated.
